# Supplementary material for: Sex-Specific Associations between Blood Pressure and Risk of Atrial Fibrillation Subtypes in the Tromsø Study
Source: J Clin Med. 2021 Apr 5;10(7):1514. doi: 10.3390/jcm10071514 (PMC8038622; doi:10.3390/jcm10071514)
Supplement: Supplementary file 1 [file jcm-10-01514-s001.zip › Supplementary Data - Table S3.docx]

**Supplementary Data – Table S3**

**Table S3.** Sex-specific means of systolic blood pressure (mmHg) by AF subtype and by hypertension group (The Tromsø Study).

|  | **Women** | |  | **Men** | |
| --- | --- | --- | --- | --- | --- |
|  | **Parox/pers AF** | **Perm AF** |  | **Parox/pers AF** | **Perm AF** |
| **HT group^a^** | **Mean** | **Mean** |  | **Mean** | **Mean** |
| Normotensive | 121.36 (9.54) | 122.46 (9.34) |  | 127.62 (7.97) | 126.21 (8.81) |
| Controlled HT | 128.26 (8.52) | 131.23 (6.16) |  | 129.40 (7.49) | 128.43 (8.64) |
| Uncontrolled HT | 172.66 (21.57) | 165.10 (20.91) |  | 166.67 (18.83) | 162.43 (19.08) |
| Untreated HT | 158.44 (17.42) | 159.87 (18.59) |  | 153.45 (17.31) | 152.76 (16.43) |

AF indicates atrial fibrillation; HR, hazard ratio; CI, confidence interval; HT, hypertension; parox, paroxysmal; pers, persistent; perm, permanent. Values are mean (SD); the means are adjusted for age using linear regression model.

^a^Normotensive: SBP<140 mmHg and DBP<90 mmHg and no current antihypertensive medication use. Controlled hypertension: SBP<140 mmHg and DBP<90 mmHg and current antihypertensive medication use. Uncontrolled hypertension: SBP≥140 mmHg and/or DBP≥90 mmHg and current antihypertensive medication use. Untreated hypertension: SBP≥140 mmHg and/or DBP≥90 mmHg and no current antihypertensive medication use.
