# Supplementary material for: Sex-Specific Associations between Blood Pressure and Risk of Atrial Fibrillation Subtypes in the Tromsø Study
Source: J Clin Med. 2021 Apr 5;10(7):1514. doi: 10.3390/jcm10071514 (PMC8038622; doi:10.3390/jcm10071514)
Supplement: Supplementary file 1 [file jcm-10-01514-s001.zip › Supplementary Data - Table S1.docx]

**Supplementary Data – Table S1**

|  | **Women *n* = 12,488** | | | |  | **Men *n* = 10,830** | | | |
| --- | --- | --- | --- | --- | --- | --- | --- | --- | --- |
| **Systolic blood pressure, mmHg** | **Parox/pers AF** | | **Permanent AF** | |  | **Parox/pers AF** | | **Permanent AF** | |
|  | **HR** | **95% CI** | **HR** | **95% CI** |  | **HR** | **95% CI** | **HR** | **95% CI** |
| **100** | 0.61 | 0.51, 0.72 | 0.60 | 0.46, 0.79 |  | 0.80 | 0.72, 0.88 | 0.84 | 0.76, 0.94 |
| **110** | 0.80 | 0.73, 0.86 | 0.80 | 0.71, 0.90 |  | 0.89 | 0.85, 0.94 | 0.92 | 0.87, 0.97 |
| **120** | 1.00 | ref | 1.00 | ref |  | 1.00 | ref | 1.00 | ref |
| **130** | 1.21 | 1.13, 1.30 | 1.19 | 1.08, 1.30 |  | 1.12 | 1.06, 1.18 | 1.09 | 1.03, 1.15 |
| **140** | 1.43 | 1.26, 1.63 | 1.36 | 1.15, 1.60 |  | 1.25 | 1.13, 1.38 | 1.19 | 1.07, 1.32 |
| **150** | 1.65 | 1.38, 1.97 | 1.51 | 1.21, 1.89 |  | 1.40 | 1.20, 1.62 | 1.29 | 1.10, 1.51 |
| **160** | 1.87 | 1.50, 2.34 | 1.65 | 1.26, 2.17 |  | 1.56 | 1.28, 1.91 | 1.41 | 1.14, 1.74 |
| **170** | 2.09 | 1.61, 2.72 | 1.78 | 1.30, 2.43 |  | 1.75 | 1.36, 2.24 | 1.53 | 1.18, 1.99 |
| **180** | 2.31 | 1.72, 3.11 | 1.89 | 1.34, 2.67 |  | 1.95 | 1.45, 2.63 | 1.67 | 1.21, 2.29 |
| **AF cases, n** | 395 | | 325 | |  | 477 | | 378 | |
| **P value** | <0.001 | | <0.001 | |  | <0.001 | | 0.002 | |

**Table S1.** Hazard ratios for the association between systolic blood pressure and AF subtypes in women and men when excluding participants with one or more of the comorbidities (myocardial infarction, angina pectoris, stroke, and diabetes mellitus) from the study sample (The Tromsø Study).

AF indicates atrial fibrillation, HR, hazard ratio; CI, confidence interval; parox, paroxysmal; pers, persistent. HRs and 95% CIs were calculated using AF subtype as the dependent variable and fractional polynomials of SBP as the main exposure using SBP of 120 mmHg as the reference value, and are adjusted for age, body mass index, current smoking, total cholesterol and leisure time physical activity.
